# Red Beet Process Waste: A Sustainable Glucose Syrup Alternative for Gummy Confectionery

**DOI:** 10.1111/1750-3841.70262

**Published:** 2025-05-07

**Authors:** Tahra ElObeid, Burcu Tuzun, Ayşe Apaydin, Ezgi Tekneci, Ibrahim Palabiyik, Omer Said Toker, Nevzat Konar, Ilyas Atalar

**Affiliations:** ^1^ Department of Nutrition Sciences, College of Health Sciences, QU Health Qatar University Doha Qatar; ^2^ Agriculture Faculty, Food Engineering Department Eskişehir Osmangazi University Eskisehir Turkey; ^3^ Independent Researcher Istanbul Turkey; ^4^ Agriculture Faculty, Food Engineering Department Tekirdag Namik Kemal University Tekirdag Turkiye; ^5^ Faculty of Chemical and Metallurgical Engineering, Food Engineering Department Yildiz Technical University Istanbul Turkey; ^6^ Agriculture Faculty, Dairy Technology Department Ankara University Ankara Turkey

**Keywords:** colorings, confectionery, corn syrup, red beet, waste treatment

## Abstract

Red beet (RB) is a key component in the natural colorant market. Various efficiency challenges, arising from process waste, affect their production. Disposal of these wastes elevates production costs and poses environmental risks. Innovative approaches have been explored to repurpose process waste to align with the circular economy. A rising trend in food technology is substituting corn syrup with clarified fruit juices or concentrates. This study aims to develop an alternative to glucose syrup from fluid waste (red beet liquid waste; RBLW) (∼12.0°Bx), which is released after pigment extraction from RB (*Beta vulgaris* L.), and to determine its potential application in gummy formulations. This study examined the clarification and decolorization of samples using ion exchange and adsorbent resins at varying flow rates, followed by evaporation. Composition and visual properties were analyzed. Under optimal conditions, the clarified and decolorized residue (clarified and decolorized red beet liquid waste [CD‐RBLW]) showed a high T625 value (77.3%) and was incorporated in gummy formulations. A Custom Mixture Design (*n* = 14) tested CD‐RBLW, glucose syrup, and gelatin as independent variables. In addition, gummy samples were stored under accelerated shelf‐life conditions (25°C/70% relative humidity [RH]) for 8 weeks, and changes in hardness, color difference (∆*E*), moisture content, and water activity values were monitored. After processing, RBLW demonstrated functionality comparable to glucose syrup in gummy formulations.

**Practical Application**: This research shows that liquid waste from RB processing can be turned into a sustainable alternative to glucose syrup, especially for making gummy candies. Clarifying and decolorizing waste makes it food‐grade, cuts costs and impact, and supports a circular economy.

## 1 Introduction

In recent years, research on reusing food processing by‐products has increased. One of the main focus areas of these studies is the utilization of fruit and vegetable by‐products and waste materials. Some recent examples of studies examining such materials include pomegranate fruit peel (Motlatsi et al. 2024), tamarillo wastes and by‐products (Machado et al. 2024), red cabbage wastes (Oliveira et al. 2025), date by‐products (Musa and Elnour 2024), mango bagasse (Luzardo‐Ocampo et al. 2025), nut by‐products (Alasalvar et al. 2024), citrus by‐products (da Silva et al. 2024), and cashew apple bagasse (Zie et al. 2025). Despite being a significant natural pigment source, red beet (RB) (*Beta vulgaris* L.) juice by‐products and waste have received relatively little attention in reuse studies compared to other industrial food by‐products (Atalar et al. 2024; Costa et al. 2017). Given the importance of natural colorants and the widespread use of beet‐based pigments, further research into reusing process waste is essential. Additionally, most other plant waste does not originate from industrial production processes. However, RB waste is generated in increasing amounts due to the growing consumer demand for natural colorants (Ghosh et al. 2022; Thomsen et al. 2023). Moreover, the direct consumption of this waste as food or a food ingredient is limited. Therefore, it is important to emphasize the significance of studies focused on the reutilization of solid and liquid waste generated during the RB colorant production process.

RB extract, a rich source of betalains, is a key component in the natural colorant market. (Sadowska‐Bartosz and Bartosz 2021). However, extracting high‐betalain content from *B. vulgaris* is typically inefficient. RB juice has a high sugar content (Trishitman et al. 2021). Consequently, process liquid waste released after pigment extraction also has a high sugar content. This situation poses a disadvantage in treatment but increases the potential for reuse. Industrial process fluid wastes have a protein content of approximately 10.0 g/100 g (in dm), whereas the amounts of mono‐ (glucose and fructose) and disaccharides (sucrose) are >70.0 g/100 g (in dm) (Atalar et al. 2024). Consequently, the main components of the sugar profile in RB juice consist of sucrose, glucose, and fructose. The balance between the amounts of monosaccharides in this composition, along with the significant presence of sucrose (Trishitman et al. 2021; Atalar et al. 2024), provides an important sweetness advantage for RB juice compared to glucose syrup, whose primary mono‐ and disaccharide is glucose (Hartel et al. 2018). This is because the sweetness index of glucose is lower than that of other sugars (Gok et al. 2020).

There are very limited studies investigating the potential use of liquid waste generated after pigment extraction in the natural coloring process within food technology (Atalar et al. 2024; Costa et al. 2017). No study has been found on the clarification, decolorization, and/or subsequent reuse of the waste from RB juice pigment extraction in food technology. RB juice has a high sugar content (Trishitman et al. 2021). The waste from the RB coloring process also has significant potential in terms of sustainability and circular economy approaches in food technology due to its high soluble solids content (approximately 12 °Brix) and the fact that a significant portion of these soluble solids consists of mono‐ (glucose and fructose) and disaccharides (sucrose). To the best of our knowledge, this study is the first to explore the reuse of this waste material by incorporating it into gummy confectionery formulations after clarification and decolorization. Additionally, interactions with gelatine and corn syrup (42 dextrose equivalent [DE] glucose syrup) were investigated.

Red beet processing liquid waste (RBLW) presents a promising alternative due to its high content of mono‐ and disaccharides. However, before it can be effectively utilized, pre‐treatment processes such as clarification and decolorization are required to eliminate residual pigments and other impurities. These residues limit the usage possibilities and areas of RB process liquid wastes. This is because the visual properties of sugar syrups, particularly color and clarity, are considered indicators of quality and sensory attributes (Henke et al. 2019). The commonly used color removal method for sugar syrups and fruit juices is ion exchange and adsorbent resins. Fundamental properties to consider for removing color molecules from the main matrix include molecular weight, polarity, and degree of ionization. The pH value of the medium must be considered for the ionization of a molecule (Li et al. 2019). Suitable ion exchange resins are porous, insoluble polymers with high surface area. They selectively separate target components from the matrix at relatively high temperatures and different pH levels (Henke et al. 2019). Additionally, color removal allows for the use of syrups in various applications.

This study aims to develop and characterize a material obtained by passing fluid wastes released after the extraction process in the industrial RB coloring process, which contains residual pigments. Fluid waste decolorization and clarification were performed using anion, cation exchange, and adsorber resin. Additionally, the study aims to determine the potential use of the obtained glucose syrup alternative material in gummy candies and to investigate its effects on shelf life and final product stability. There are various studies related to the use of RB‐derived colorants in various confectionery products, including gummies. However, to the best of our knowledge, no prior research has reported the clarification and decolorization of RBLW and the use of the obtained material in any confectionery product. This highlights the innovative and original aspect of our study.

## 2 Materials and Methods

### 2.1 Materials

In the preparation of gummy samples, water, sucrose (Konya Sugar, Konya, Turkey), 40–42 DE glucose syrup (Sunar, Adana, Turkey), and 220–250 bloom food grade bovine gelatin (Gerede Jelatin, Bolu, Turkey) were used. RBLW was obtained from an industrial coloring agent producer (X Company, Kocaeli, Turkey). Cation exchange resin (Macro‐Prep 25S, Bio‐Rad, Turkey), anion exchange resin (Macro‐Prep, High Q, Bio‐Rad, Turkey), and two different adsorbers (Modified Styrene‐ divinylbenzene (DVB), 600.0 m^2^/g, 1.18 g/mL, and Crosslinked polystyrene, 600.0 m^2^/g, 1.02 g/mL) (Merck, Darmstadt, Germany) were used in the clarification and decolorization of RB extraction process liquid waste.

### 2.2 Study Design

This study was conducted in two stages. In the first stage, after the evaporation (> 65.0°Bx) of RBLW (∼12.0°Bx), which is released after pigment extraction from RB juice concentrate in the production of industrial RB extract powder, clarification and decolorization studies were carried out by passing it through anion and cation exchange resins and different adsorbent resins at three different flow rates. After the characterization of the samples, the conditions under which clarified and decolorized red beet liquid waste (CD‐RBLW) with the highest clarification value T625 (transmittance values of the samples at 625 nm) of 77.3% was determined. In the second stage, gummy samples were prepared with CD‐RBLW, glucose syrup, and gelatin amounts as independent variables under the conditions determined in the first stage, and an RSM study was conducted. The gummy samples were characterized, and their stability (color, texture, water activity, and moisture content) behaviors were investigated under accelerated shelf‐life (ASL) conditions (25°C/70% relative humidity [RH]).

### 2.3 Clarification and Decolorization of RB Extraction Process Liquid Waste

This study utilized liquid waste samples from the extraction process of RB (*B. vulgaris* L.) grown in Eregli Province (Konya, Türkiye). The liquid material with a soluble solid content of ∼12.0°Bx (20.0 ± 5.00°C, pH < 3.50) was released after pigment extraction from juice concentrate in the production of RB extract powder (Eregli, Turkey) by a local company (Kocaeli, Turkey) was used as RBLW (Supporting Information File 1).

On the basis of various preliminary studies conducted for clarification and color removal, optimal conditions for anion and cation resins were determined. This study used the type of adsorbent resin and the flow rate as independent variables. Considering the bed height of the resins (60–66 cm), samples were transferred to columns with an inner diameter of 4 cm for the clarification and color removal of RBLW. These samples were then subjected to procedures established in the aforementioned preliminary studies. Initial pH values were determined as < 3.50. Subsequently, samples were passed through a cation exchange resin at 2 BV/h and pH values increased to 8.00–8.20. Then, samples were passed through an anion exchange resin at the same flow rate. Samples with a pH of < 3.5 were then subjected to color removal using an adsorber resin. During this process, three different flow rates (1.00, 1.5, and 2.00 BV (bed volume/h) and two different adsorbers (Modified Styrene‐DVB and Crosslinked polystyrene) were used. Subsequently, the samples underwent a second cation exchange resin treatment at 2 BV/h. The clarified and decolorized samples were evaporated to a soluble content of at least 65°Bx (65°C, 600 mmHg vacuum, pilot‐type evaporator, Company X, Kocaeli, Turkey). At least 500 mL of all samples were prepared under the same conditions and stored in previously sterilized glass containers in a dark environment at 4°C until analysis. The adsorber type and flow rate at which the highest degree of clarification (Transmittance 625 nm) was achieved in the obtained RBLW were identified. Additionally, in samples prepared under all conditions (*n* = 6), degrees of clarification, Brix, pH, and total acidity values, color properties (*L* ^*^, *a* ^*^, *b* ^*^, chroma, and hue angle), browning index (A420 nm), total phenolic content, crude protein, total ash, total sugar, sucrose, glucose, and fructose amounts were determined. In the preparation of gummy samples, which served as a model food in the study, the CD‐RBLW with the highest T625 nm value (77.3% ± 3.58%) and a flow rate of 1.5 BV/h using Modified Styrene DVB was used as an alternative to glucose syrup (Table 1).

**TABLE 1:** Properties clarified and decolorized red beet process liquid waste prepared by using various adsorber and flow rate.

Parameter	A1			A2		
	1.0 BV/h	1.5 BV/h	2.0 BV/h	1.0 BV/h	1.5 BV/h	2.0 BV/h
pH	3.67 ± 0.02^c^	3.32 ± 0.01^d^	3.71 ± 0.03^c^	4.21 ± 0.11^a^	4.09 ± 0.32^b^	3.74 ± 0.94^c^
TSS (°Bx)	65.2 ± 1.21^b^	66.3 ± 0.23^b^	64.4 ± 0.95^b^	68.3 ± 1.11^a^	69.1 ± 0.34^a^	70.2 ± 0.29^a^
TAC (g/100 g)	6.21 ± 0.21^a^	5.05 ± 0.01^d^	6.12 ± 0.33^a^	5.55 ± 0.32^c^	5.93 ± 0.75^b^	6.21 ± 0.56^a^
TPC	77.3 ± 3.51^c^	58.1 ± 0.83^e^	69.1 ± 4.21^d^	81.1 ± 1.15^b^	84.3 ± 2.23^a^	87.2 ± 0.56^a^
CP (g/100 g)	4.11 ± 0.45^c^	3.10 ± 0.41^d^	4.25 ± 0.11^c^	5.56 ± 0.21^b^	6.43 ± 0.19^a^	6.81 ± 0.75^a^
*L* ^*^	69.2 ± 1.75^b^	76.6 ± 2.32^a^	67.5 ± 3.01^b^	63.3 ± 2.33^c^	62.1 ± 0.42^c^	60.2 ± 0.35^d^
*a* ^*^	3.21 ± 0.24^a^	−1.71 ± 0.90^c^	2.19 ± 0.41^b^	2.33 ± 0.05^b^	3.12 ± 0.03^a^	2.45 ± 0.07^b^
*b* ^*^	48.3 ± 4.11^b^	52.9 ± 7.12^a^	50.4 ± 3.21^a^	51.2 ± 0.89^a^	50.5 ± 0.42^a^	44.3 ± 2.35^b^
*C* ^*^	49.1 ± 5.51^b^	52.8 ± 7.10^a^	51.3 ± 2.75^a^	52.1 ± 1.11^a^	51.7 ± 0.32^a^	48.5 ± 0.15^b^
*h*°	87.9 ± 2.11^c^	91.9 ± 1.07^b^	89.1 ± 2.01^c^	95.0 ± 3.21^a^	94.2 ± 2.17^a^	90.9 ± 2.45^b^
CD (T625, %)	66.3 ± 2.34^c^	77.3 ± 3.58^a^	72.1 ± 1.12^b^	65.3 ± 2.56^c^	67.1 ± 3.01^c^	62.3 ± 4.16^d^
BI (A420)	0.093 ± 0.005^b^	0.092 ± 0.003^b^	0.093 ± 0.005^b^	1.397 ± 0.004^a^	1.398 ± 0.007^a^	1.403 ± 0.009^a^
TA (g/L)	0.76 ± 0.02^b^	0.63 ± 0.03^b^	0.71 ± 0.09^b^	1.02 ± 0.03^a^	0.98 ± 0.01^a^	1.04 ± 0.05^a^
TS (g/100 g)*	64.3 ± 2.05^c^	65.21 ± 2.35^c^	66.7 ± 1.11^b^	72.3 ± 3.67^a^	68.2 ± 2.69^b^	71.3 ± 3.21^a^
AA (EC50, %)	19.5 ± 0.03^b^	20.2 ± 0.01^b^	21.3 ± 0.02^b^	20.1 ± 0.09^b^	19.5 ± 0.17^b^	23.0 ± 1.29^a^
*S* (g/100 g)*	14.3 ± 0.21^a^	12.5 ± 1.04^b^	15.5 ± 2.10^a^	16.2 ± 1.15^a^	15.5 ± 1.24^a^	16.9 ± 1.96^a^
*G* (g/100 g)*	22.5 ± 2.09^b^	24.8 ± 3.64^a^	25.1 ± 1.17^a^	26.3 ± 2.12^a^	24.5 ± 0.95^a^	26.3 ± 3.29^a^
*F* (g/100 g)*	23.9 ± 0.94^c^	25.3 ± 1.87^b^	27.1 ± 2.15^a^	28.2 ± 1.10^a^	27.3 ± 2.06^a^	29.1 ± 3.35^a^

*Note*: A1: Modified Styrene DVB (600 m^2^/g, 1.18 g/mL), A2: Crosslinked polystyrene (600.0 m^2^/g, 1.02 g/mL), Fructose, *F* mean ± standard deviation. Different lowercase letters indicate a significant difference in the values of the samples in the same row depending flow rate and adsorber. *p* >  0.05.

Abbreviations: AA, antioxidant activity; BI, browning index; CD, clarification degree; CP, crude protein; G, glucose; S, sucrose; TA, total acidity; TAC, total ash content; TS, total sugar; TPC, total phenolic compounds in mg GAE/kg; TSS, total soluble solids.

*in dry matter.

### 2.4 Analysis of Clarified and Decolorized Liquid Wastes

#### 2.4.1 Physicochemical Analysis

The pH values of RBLW samples were measured using a digital pH meter (PB‐10, Sartorius, Germany). This measurement was conducted during preliminary trials for all sample groups before and after each resin application and before and after evaporation. The pH values after evaporation were determined in the clarification and color removal studies. The total solid content of the samples after evaporation was measured in Brix (°Bx) using a handheld automatic digital refractometer (PAL, Atago, Japan). The ash content of the samples before and after evaporation, as an indicator of the inorganic residue remaining after complete combustion, was determined as a percentage (m/m) of ash content. For this purpose, 10 g of the sample was transferred to a pre‐weighed and dried crucible and then placed in an ash furnace at 500°C for 3 h. After combustion, the samples were cooled in a desiccator and weighed to determine the ash content. Total acidity was determined using a 0.2 M sodium hydroxide solution and phenolphthalein indicator, with the results calculated in g citric acid equivalent/L.

#### 2.4.2 Clarity and Color

The aim is to determine browning caused by compounds that may be released as a result of various reactions, primarily Maillard reactions, in which reducing sugars present in the composition participate. For this purpose, the absorbance value measured at a wavelength of 420 nm is used as the main criterion (Li et al. 2019). Another critical indicator is clarity. Spectrophotometric analyses are among the frequently used methods for clarity measurements. These methods are based on converting the absorbance at a specific wavelength into a transmittance value and making an evaluation on the basis of this ratio. The commonly used wavelength for glucose syrups is 600 nm, and the determined clarity value is referred to as T600 (Djalal et al. 2019; Morales et al. 2024). This study aimed to obtain a material that could be used as a glucose syrup substitute, such as clarified and decolorized apple juice concentrate. In studies on clarified apple juice concentrates, measurements were performed at a wavelength of 625 nm, and the T625 value was used for clarity evaluation.

The clarity of the samples was determined using the spectrophotometric method (Zhao et al. 2014). For this purpose, the absorbance and transmittance values of the samples at 625 and 420 nm after evaporation were measured using a UV–Vis spectrophotometer (Jasco UV/Vis Spectrophotometer, Japan). Color properties of samples and commercial 42 DE glucose syrup (*L* ^*^, *a* ^*^, *b* ^*^, chroma, and hue angle) were determined using a colorimeter (Chroma Meter CR‐400, Konica Minolta, Japan).

#### 2.4.3 Sugar Profile

Total sugar content was determined using a titrimetric method with Fehling solutions. After evaporation, the sugar profile (sucrose, fructose, and glucose contents) of the RBLW samples was determined using an HPLC system with a refractive index detector. For this purpose, an Aminex HPX‐87H column (300 × 7.8 mm^2^) (Bio‐Rad, Istanbul) was used at 55°C, with a flow rate of 0.3 mL/min and a mobile phase of 6% acetonitrile and 0.045 N H_2_SO_4_ (Kelebek et al. 2009).

#### 2.4.4 Crude Protein

The crude protein content (*N* × 6.25) was determined by the Kjeldahl Method (Atalar et al. 2024).

#### 2.4.5 Total Phenolic Contents

To determine total phenolic contents, 40 µL of the sample diluted with methanol (1:10) was placed into a spectrophotometer cuvette (macro). A volume of 3.16 mL of distilled water and 200 µL of Folin–Ciocalteu separating solution (Merck, Darmstadt, Germany) were added. After waiting 1–2 min, 600 µL of 20% Sodium Carbonate solution (Merck, Germany) was added. The mixture was left to stand at room temperature for 2 h. Then, absorbance values at 765 nm wavelength against a blank were read on a spectrophotometer (Jasco UV/Vis Spectrophotometer, Japan). The calculation was performed using a calibration curve of gallic acid.

#### 2.4.6 Antioxidant Activity Potential

The extract reacted (100 µL) with DDPH solution (3900 µL) for 1 h in the darkness, and absorbances were measured at 517 nm (Jasco UV/Vis Spectrophotometer, Japan). The concentration that would provide at least 50% inhibition in DPPH was calculated (Equation 1) as the inhibition concentration (Demirci et al. 2024).

(1) $$\mathrm{Inhibition}\left(\%\right)=\left[\left(A_{C}-A_{T}\right)/A_{C}\right]\times 100$$

where *A*_c_ is control absorbance value, *A*_t_ is sample absorbance value.

### 2.5 Model Food Study: Gummy

#### 2.5.1 Sample Preparation

Model gummy samples were modified from the composition, color, and aroma properties used by Gok et al. (2020) to determine the effects of alternative glucose syrup substitutes. Water, sucrose, 42 DE glucose syrup, gelatin, and CD‐RBLW with the highest T625 nm value were used for this aim. Clarified syrup samples were prepared using experimental designs on the basis of Response Surface Methodology Custom Mixture Design (Design Expert, ver. 13) (Table 2). For each sample group, the total feed amount was determined as 120 g, including water, in terms of dry matter, on the basis of the amount of water used to prepare the deionized sugar syrup (65°Bx), glucose syrup (80°Bx), and gelatin solution (1:2), considering the amounts of soluble solids in water. Samples were prepared using a Thermal Mixer (Thermomix TM5, Vorwerk, Germany), where water, sucrose, glucose syrup, and/or CD‐RBLW were mixed at 100°C and stirred at 200–300 rpm until reaching 85°Bx. After cooling to 90°C, samples were taken for pH and °Brix measurement. A pre‐prepared 250‐bloom gelatin‐water solution (1:2) was added and stirred at 90°C for 5 min at 200–300 rpm. Subsequently, the syrup was transferred into silicone molds and left at 20°C for 24 h. Samples from the molds were coated with starch to prevent sticking, placed in polyethylene bags, and stored at room temperature. Experiments were conducted in batches of approximately 500 g.

**TABLE 2:** Study design of gummy samples made using clarified and decolorized red beet liquid waste (CD‐RBLW) in different formulations.

Sample	Clarified and decolorized RBLW (g/100 g, in dm)	Glucose syrup (42 DE) (g/100 g, in dm)	Gelatin (g/100 g in dm)	Sucrose (g/100 g in dm)
1	41.00	0.00	5.70	42.27
2	41.00	0.00	5.70	42.27
3	20.13	20.13	6.45	42.27
4	27.57	14.13	5.00	42.27
5	0.00	39.70	7.00	42.27
6	39.70	0.00	7.00	42.27
7	0.23	41.00	5.47	42.27
8	0.23	41.00	5.47	42.27
9	10.20	30.05	6.45	42.27
10	13.23	26.47	7.00	42.27
11	26.47	13.23	7.00	42.27
12	20.85	20.85	5.00	42.27
13	20.13	20.13	6.45	42.27
14	30.05	10.20	6.45	42.27

*Note*: For each sample group, the amount of water‐soluble dry matter of deionized sugar syrup (65°Bx) and glucose syrup (80°Bx) and the amount of water used to prepare the gelatin solution (1:2) were considered in the total input mass in terms of dry matter and based on a feed amount of 120 g including water.

Abbreviation: DE, dextrose equivalent.

#### 2.5.2 Moisture Content

The moisture content of the samples was determined using a gravimetric technique by placing them in an oven (Memmert UF110, Schwabach, Germany)) at 60°C until a constant weight was achieved (Periche et al. 2015).

#### 2.5.3 Water Activity

The water activity values of the samples were determined using a water activity analyzer (LabMaster aw, Novasina, Lachen, Switzerland) at 20.00°C ± 2.00°C.

#### 2.5.4 pH

The pH values of the candy samples were determined using a pH meter (ST400, Mettler Toledo, Istanbul, Turkiye) in the initial syrup (Periche et al. 2014).

#### 2.5.5 Color

The color values of the samples (*L* ^*^, *a* ^*^, *b* ^*^, chroma, and hue angle) were determined using a colorimeter device (Chroma Meter CR‐400, Konica Minolta, Japan).

#### 2.5.6 Texture Profile Analysis (TPA)

After preparation with 28 mm diameter and 20 mm height dimensions, the candy samples were subjected to TPA using a TA‐TX plus instrument (Stable Micro Systems, Godalming, UK). The device is equipped with a 5 kg load cell and a cylindrical probe with a diameter of 35 mm. Compression at 50% was applied twice consecutively for 15 s each, with a test speed of 1 mm/s. Hardness, springiness, adhesiveness, gumminess, chewiness, and resilience values were determined using the force‐time curves. The analyses were conducted with at least five replicates.

#### 2.5.7 Total Phenolic Content

The method was described in Section 2.4.

#### 2.5.8 Fourier Transform‐Infrared (FT‐IR) Spectra

Spectral measurements were performed within the range of 4000–650 cm^−1^. All measurements were performed in triplicate with a diamond triple‐bounce ATR accessory (Bruker Alpha, Germany)

#### 2.5.9 ASL Study

Following their preparation, the samples were placed in a shelf‐life cabinet without packaging and subjected to ASL testing conditions for samples held at 25°C and 70% RH (Subramaniam 2016). At 7‐day intervals, color analysis, TPA, and water activity were conducted. *L* ^*^ (brightness), *a* ^*^ (±red‐green), and *b* ^*^ (±yellow‐blue) values were measured, and Δ*E* ^*^ values were determined on the basis of Equation (2). Δ*L* ^*^, Δ*a* ^*^, and Δ*b* ^*^ values were calculated on the basis of the initial measurement.

(2) $$\Delta E^{*}={\left[{\left(\Delta L^{*}\right)}^{2}+{\left(\Delta a^{*}\right)}^{2}+{\left(\Delta b^{*}\right)}^{2}\right]}^{1/2}$$

Moreover, Δ*aw* and ΔHardness values were determined by considering initial and last analysis results at 49 days of ASL. For this aim, the difference between the initial and last analysis results was used to determine the variance percentage (%).

### 2.6 Statistical Analysis

RSM Custom Mixture Design was used to evaluate the effects of CD‐RBLW, glycose syrup, and gelatine concentrations on the gummy formulation. A total of 14 experiments were designed using Design Expert software (Stat‐Ease Inc. Trial version 13.0, Minneapolis, USA). Statistical analyses were conducted with a confidence interval of 95% and performed three times. The design is provided in Table 2. The second‐order polynomial model was proposed for each response variable (Equation 3).

(3) $$\begin{array}{ccc} Y & = & b_{0}+b_{1\;}X_{1}+b_{2\;}X_{2}+b_{3}X_{3\;}+b_{11\;}X_{1}X_{1}+b_{22\;}X_{2}X_{2\;}+b_{33\;}X_{3}X_{3}\\ & & +b_{12\;}X_{1}X_{2}+b_{13\;}X_{1}X_{3}+b_{23\;}X_{2}X_{3} \end{array}$$

where *b*_0_ is the intercept; *b*_1_, *b*_2_, and *b*_3_ were linear; *b*_11_, *b*_22_, and *b*_33_ are quadratic; *b*_12_, *b*_13_, and *b*_23_ are interaction terms; and *X*_1_, *X*_2_, and *X*_3_ are the independent variables.

The results of the analyses were expressed as mean ± standard deviation. The data obtained from the preparation of CD‐RBLW samples in the study were subjected to one‐way analysis of variance (ANOVA). ANOVA variance analysis was performed to determine the statistical significance of the analysis results. Statistical analyses were conducted with a confidence interval of 95%.

## 3 Results and Discussion

### 3.1 RBLW Clarification and Decolorization

The transmittance value T625 nm of RBLW samples obtained after clarification and decolorization processes was determined to be 77.3% ± 3.58% (Table 1). According to this result, Modified Styrene‐DVB (600.0 m^2^/g, 1.18 g/mL) was used as an adsorber at 1.00 BV/h. The T625 value indicates the degree of clarity of fruit juices, where higher values are desired for more apparent samples (Li et al. 2019). The target value for clarified fruit juices and concentrates is around 95.0% (Zhao et al. 2014). Although not sufficiently addressed in scientific studies, the production and supply of clarified, decolorized, and deionized fruit juice concentrates have become widespread worldwide in recent years. These products are used as alternative and natural sweeteners in the composition of various foods. The most commonly used fruit for this purpose is apple. Therefore, our study also considers previous research conducted with apple juice concentrate. Additionally, this study aimed to obtain a material with high potential to substitute commercial glucose syrups. As previously emphasized, clarity is one of the key criteria for sugar syrup substitutes (Henke et al. 2019). This is because increased clarity and neutral color properties enhance the potential for use in various food formulations. The transmittance value (T600), determined on the basis of the absorbance at a wavelength of 600 nm, is used to define the clarity characteristics of glucose syrups. In a previous study utilizing activated carbon, T600 values for glucose syrup were reported to be between 67.9% and 70.16% (Djalal et al. 2019). Therefore, we can conclude that the clarity values determined for RBLW are acceptable.

Although, in the production of apple juice concentrate, the T625 value was 56.94% after mashing, whereas it reached 81.14% with enzymatic clarification and 96.33% after ultrafiltration (UF), resin decolorization, and concentration processes (Li et al. 2019). However, these products typically have a lower raw protein content. Both the presence and denaturation of proteins can promote turbidity. Although apple juices typically have higher pH values (> 3.60) (Li et al. 2019), the decolorized and clarified RBLW samples have a lower value (3.32 ± 0.01). In the study investigating the decolorization efficiency of eight different activated carbons obtained from sugar beet pulp and two commercial activated carbons in sugar syrup, it was reported that the decolorization efficiency of sugar beet pulp activated carbon prepared with 750°C temperature and 5 h CO_2_ application was close to commercial activated carbons. It was determined that approximately 60% of the color substances were removed (Mudoga et al. 2008). In the study in which activated carbon was used at different conditions and concentrations to remove dark pigments in date syrup, the maximum removal of colored components was 95% at 60°C (Nasehi et al. 2012). Al‐Farsi (2003) investigated five different treatments for clarification of date syrup and reported that activated carbon, along with filtration, gives the highest decolorization and leaves the lowest ash.

The A420 nm value is considered an index of browning. The decrease in this value can be attributed to removing or preventing pigments, which are products of browning reactions. For apple juice and concentrate, this value was 0.42 before UF and resin color removal and subsequently decreased to 0.06 and 0.04, respectively. In our study, the A420 nm value for RBLW samples was 0.09 after processing. Consequently, the browning index and transmittance values indicate that materials comparable to clarified apple juice and concentrate were obtained. However, the *L* ^*^ (76.6 ± 2.32) and *a* ^*^ (−1.71 ± 0.90) values were lower than those of UF apple juice (Zhao et al. 2014), and the *b* ^*^ value (52.9 ± 7.12) was higher (Table 1).

### 3.2 RBLW Characterization

#### 3.2.1 Physicochemical Properties

The food industry highly values fruit juice concentrates due to their elevated sugar and soluble solids content, typically measured in degrees Brix (°Bx). This measurement reflects the amount of dissolved sugar and is a critical quality indicator, as it directly impacts sweetness, texture, and preservation qualities. Standards for fruit juice concentrates often require at least 65.0 °Bx to ensure sufficient sweetness and viscosity. Commercial glucose syrups, such as those with a 42 DE, are even more concentrated, with typical values around 80.0 °Bx, adding sweetness and bulk in a range of food products (Pekdogan Goztok et al. 2022). Additionally, the lower soluble solid content in commercial high‐fructose corn syrups (HFCS) (71%–77%) (Helstad 2018) and the presence of sucrose and fructose in the composition of RBLW, alongside glucose, should be taken into account when evaluating our findings.

As a by‐product of beet processing, RBLW presents a promising alternative concentrated source. Following clarification, color removal, and evaporation (to concentrate sugars), RBLW samples reached a Brix value of 66.3 ± 0.23 °Bx. This level not only meets but exceeds the minimum threshold of 65.0 °Bx, aligning RBLW with conventional fruit juice concentrates. This process indicates that RBLW can achieve a level of sweetness and density suitable for commercial applications, potentially providing a lower‐cost alternative to traditional concentrates like apple juice, which is commonly used in the industry as a base or sweetener.

#### 3.2.2 Sugar Profile and Functional Comparisons

Sugar type and concentration are important parameters affecting the texture, shelf life, and microstructure of gummy‐style candies. In addition to these properties of sugars, their interaction with other sugars in the formulation is also an important parameter regarding product quality. For this purpose, it is important to determine the sugar profile of sugar‐containing ingredients to be used in the formulation of gummy production. In terms of sugar composition, the RBLW concentrate contains a mix of sucrose (12.5 ± 1.04 g/100 g, dm), glucose (24.8 ± 3.64 g/100 g, dm), and fructose (25.3 ± 1.87 g/100 g, dm), resulting in a total sugar concentration of 65.2 ± 2.35 g/100 g. This composition indicates that RBLW offers a balanced mix of monosaccharides (glucose and fructose) and disaccharides (sucrose), supporting immediate and sustained energy release. The sugar profile of 40 DE corn syrup, which is widely used in confectionery technology, is quite different from that of RBLW. In these commercial products, sucrose is not present in the composition, and the total sugars consist on average of 20% dextrose, 19.7% maltose, 38.9% dextrins, and 21.4% higher sugars (Helstad 2018). However, the high levels of glucose and fructose are similar to other fruit‐based concentrates, which are also rich in simple sugars. Although apple juice concentrate often has a distinctive flavor profile and aroma, RBLW could provide comparable sweetness without strongly impacting flavor profiles in the final product, depending on its application. RBLW's unique sugar profile makes it a compelling alternative to fruit juice concentrates, offering versatility in confectionery, beverages, and baked goods industries increasingly prioritizing natural sweeteners over refined sugars. Additionally, using RBLW aligns with trends in upcycling agricultural by‐products, which addresses sustainability by reducing waste in food production systems.

#### 3.2.3 Ash Content

The ash content in food samples represents the total mineral residue after combustion, offering insights into their mineral composition. For the RBLW concentrate, the ash content was measured at 5.05 ± 0.01 g/100 g, which indicates a considerable mineral presence. RB often contains minerals like potassium, calcium, and magnesium. They can contribute to the nutritional value of the concentrate, making it appealing for consumers seeking functional ingredients that offer more than just sweetness. The ash content also suggests that RBLW could play a role in formulations aimed at mineral fortification, an emerging trend in health‐oriented food products. However, we must note that CD‐RBLW samples have a significantly higher total ash content than commercial corn syrups. Although it varies depending on the DE value and ion exchange processing, the total ash content in corn syrups generally ranges between 0.03% and 0.3%. The high ash content of CD‐RBLW may be a disadvantage, particularly for foam stability in aerated products (Helstad 2018).

#### 3.2.4 Total Phenolic Compounds (TPC) and Antioxidant Capacity

RB processing waste is not only a source of sugar but also rich in bioactive compounds, notably polyphenols like phenolic acids and flavonoids (Rodríguez‐Félix et al. 2022). These compounds are associated with antioxidant activities that help neutralize free radicals, potentially benefiting human health by reducing oxidative stress. In the RBLW samples analyzed, the total phenolic content was 58.1 ± 0.83 mg GAE/kg, a moderate level that could contribute to the antioxidant profile of products utilizing RBLW concentrate.

Interestingly, the total phenolic content showed an inverse relationship with the T625 value, a factor linked to the degree of thermal or processing treatment. As the T625 value increased, indicating more extensive heating or concentration, phenolic content tended to decrease. This observation aligns with the known thermal sensitivities of polyphenols, which can degrade or oxidize under high temperatures or prolonged processing, thus lowering their concentration and, consequently, their antioxidant efficacy in the final product. Moreover, the stability of betalains should be considered. Betalains are sensitive to heat and oxidation and undergo degradation during thermal processing due to isomerization and decarboxylation (Herbach et al. 2006). Additionally, factors such as sugar degradation products released during heat treatment, pH reduction, protein presence, and temperature increase accelerate the degradation rate of betalains. The optimal pH range for betalain stability is 4–6 (Trishitman et al. 2021; Manzoor et al. 2021; Prajapati and Jadeja 2022).

The antioxidant capacity of RBLW was assessed using the DPPH method, a widely used assay that measures the free radical scavenging ability of compounds. The IC50 value, the concentration required to inhibit 50% of DPPH radicals, was 20.2%, indicating moderate antioxidant activity. Although this level may be lower than some highly concentrated antioxidant sources, it is still beneficial and can contribute to the overall antioxidant profile in products using RBLW.

Antioxidants are essential in functional foods and nutraceuticals because they reduce oxidative damage associated with chronic diseases. Even at moderate levels, the natural antioxidants in RBLW enhance its potential as a functional ingredient, aligning with growing consumer interest in health and wellness. The study's findings support the viability of RBLW as a novel concentrate source for the food industry, offering a combination of soluble solids, sugars, polyphenols, and minerals. The high Brix value, favorable sugar profile, and antioxidant capacity suggest that RBLW could be a sustainable, cost‐effective alternative to traditional fruit juice concentrates. This aligns with sustainability goals by valorizing agricultural by‐products, reducing waste, and enhancing the nutritional profile of food products. Future research could focus on optimizing processing conditions to preserve polyphenolic content and enhance antioxidant capacity while maintaining the Brix level to further expand the application potential of RBLW in functional and health‐oriented foods. Additionally, the characterization of RBLW obtained after applying non‐thermal concentration techniques, such as microfiltration and UF, as well as its potential applications and effects in the composition of different foods, can be studied. Considering the use and outcomes of these technologies in fruit juice processing, it can be stated that RBLW and similar waste materials have potential for treatment applications (Lan et al. 2023; Salman et al. 2025; Ma et al. 2024).

### 3.3 Model Food Study: Gummy

#### 3.3.1 Physicochemical Properties

Tables 3 and 4 present the physicochemical properties (moisture content, pH, and water activity) of gummy samples containing CD‐RBLW, along with the modeled effects of independent variables (CD‐RBLW, glucose syrup, and gelatin concentrations) on these properties. A significant linear model was identified for the effect of independent variables on the candy samples’ pH values (4.28–5.73), with an *R* ^2^ value of 0.9919 (Table 4). The typical pH value of gummies containing food acids, especially citric acid, ranges from 3.00 to 5.00 (Burey et al. 2009; Wang and Hartel 2022). For example, Ge et al. (2021) reported this value as 3.15–5.00. Wang and Hartel (2022) determined this value as 5.00–5.30 for food acid‐free samples. It was particularly observed that the interaction between CD‐RBLW and glucose syrup affected pH. The pH values of corn syrups range from 3.75 to 5.20 (Helstad 2018). Increasing the proportion of CD‐RBLW (pH 3.32) in the formulation significantly lowered the pH value (*p* < 0.05). Acid regulators are frequently used in gummy formulations, with citric acid being the most used (Wang and Hartel 2022). However, this study did not use citric acid to observe the effect of CD‐RBLW. The pH value is important for the candy mass's quality and stability characteristics. It was concluded that using CD‐RBLW would limit the need for acid regulators. However, the potential impact of low pH on sucrose inversion and the resulting final product with a low Tg (glass transition temperature) value, which could affect the shelf life (Ergun et al. 2010), should also be considered. As a result of our study, it was found that the use of CD‐RBLW in candy technology yields similar results to the use of fruit juice concentrates as an alternative to glucose syrup (Pekdogan Goztok et al. 2022). Although the pH values of gummy candies were higher than conventional pH values, the effect of CD‐RBLW was considered a positive outcome.

**TABLE 3:** Physicochemical and color properties of gummy samples made using clarified and decolorized red beet liquid waste (CD‐RBLW) in different formulations.

Sample	TSS (°Bx)	Water activity	pH	TPC (mg GAE/kg)	Moisture content (g/100 g)	*L* ^*^	*a* ^*^	*b* ^*^	Chroma	Hue angle
1	84.5 ± 0.02	0.690 ± 0.002	4.28 ± 0.02	31.46 ± 0.02	20.19 ± 0.11	79.6 ± 1.45	−1.64 ± 0.04	11.0 ± 0.4	11.1 ± 0.40	98.6 ± 0.22
2	83.9 ± 0.01	0.690 ± 0.000	4.33 ± 0.00	45.65 ± 0.06	19.66 ± 0.52	80.0 ± 2.22	−1.62 ± 0.04	10.8 ± 0.56	10.9 ± 0.56	98.5 ± 0.47
3	86.7 ± 0.00	0.680 ± 0.001	4.88 ± 0.01	17.05 ± 0.05	15.91 ± 0.22	95.2 ± 0.26	−0.97 ± 0.04	5.51 ± 0.42	5.59 ± 0.41	100.0 ± 1.01
4	81.2 ± 0.01	0.680 ± 0.003	4.54 ± 0.00	24.39 ± 0.07	14.75 ± 0.66	76.5 ± 0.74	−1.41 ± 0.07	9.29 ± 0.53	9.40 ± 0.54	97.9 ± 1.37
5	81.5 ± 0.02	0.670 ± 0.003	5.68 ± 0.01	5.33 ± 0.10	12.37 ± 0.12	95.5 ± 0.32	−0.99 ± 0.05	6.04 ± 0.04	6.12 ± 0.03	99.3 ± 0.51
6	86.0 ± 0.01	0.690 ± 0.000	4.87 ± 0.47	37.38 ± 0.16	18.50 ± 0.08	72.3 ± 1.89	−1.57 ± 0.02	11.0 ± 0.37	11.0 ± 0.39	98.2 ± 0.29
7	86.6 ± 0.01	0.660 ± 0.004	5.66 ± 0.01	11.47 ± 0.08	12.21 ± 0.23	95.2 ± 0.47	−1.05 ± 0.08	4.81 ± 0.03	4.92 ± 0.03	102.4 ± 0.98
8	86.7 ± 0.01	0.690 ± 0.002	5.73 ± 0.01	7.23 ± 0.12	10.71 ± 0.28	97.0 ± 0.14	−0.96 ± 0.12	6.56 ± 0.24	6.63 ± 0.23	98.4 ± 1.13
9	84.8 ± 0.01	0.690 ± 0.000	5.26 ± 0.01	15.73 ± 0.16	14.80 ± 0.04	97.9 ± 0.31	−0.45 ± 0.84	6.56 ± 0.25	6.62 ± 0.25	98.2 ± 0.37
10	88.7 ± 0.01	0.680 ± 0.001	5.20 ± 0.01	13.45 ± 0.09	14.44 ± 0.01	96.7 ± 1.21	−1.03 ± 0.05	7.92 ± 0.52	7.98 ± 0.53	97.5 ± 0.12
11	85.5 ± 0.01	0.680 ± 0.003	4.80 ± 0.01	39.09 ± 0.03	17.02 ± 0.15	93.3 ± 0.64	−1.22 ± 0.06	9.11 ± 0.49	9.19 ± 0.49	97.6 ± 0.23
12	87.7 ± 0.03	0.670 ± 0.003	4.73 ± 0.00	24.92 ± 0.01	16.51 ± 0.09	85.8 ± 0.89	−1.06 ± 0.12	7.42 ± 0.90	7.50 ± 0.91	98.1 ± 0.20
13	85.1 ± 0.02	0.690 ± 0.000	4.89 ± 0.00	28.08 ± 0.08	14.85 ± 0.18	94.3 ± 0.27	−1.25 ± 0.10	8.22 ± 0.59	8.32 ± 0.59	98.7 ± 0.14
14	85.8 ± 0.01	0.660 ± 0.004	4.66 ± 0.00	39.14 ± 0.12	17.35 ± 0.04	91.5 ± 0.19	−1.18 ± 0.04	8.26 ± 0.15	8.35 ± 0.16	98.1 ± 0.14

*Note*: Mean ± standard deviation.

Abbreviations: GAE, gallic acid equivalent; TPC, total phenolic compounds; TSS, total soluble solids.

**TABLE 4:** Analysis of variance (ANOVA) results for physicochemical properties of gummy samples made using clarified and decolorized red beet liquid waste (CD‐RBLW) in different formulations.

	TSS (°Bx)		Water activity		pH		TPC (mg GAE/kg)		Moisture content (g/100 g)	
	SS	*p* value	SS	*p* value	SS	*p* value	SS	*p* value	SS	*p* value
Model	24.21	0.4080	0.0011	0.0042			1791.92	< 0.0001	91.52	< 0.0001
Lineer mix		0.4080		0.0042				< 0.0001	—	—
*X*_1_ *X*_2_	5.26	0.2956	—	—	0.1501	0.0001	1791.92			
*X*_1_ *X*_3_	0.6996	0.6940	—	—	0.0153	0.0548	228.45			
*X*_2_ *X*_3_	1.03	0.6335	—	—	0.0124	0.0777	170.37			
Lof*	32.16	0.0297	0.0007	0.0326	0.1501	0.1730	2190.74	0.8057	7.66	0.3868
P.E	1.47		0.0006		0.0153		1791.92		1.82	
Total	57.83		0.0000		0.0124		1791.92		101.00	
*R* ^2^		0.4186	*R* ^2^	0.6296	*R* ^2^	0.9919	*R* ^2^	0.8179	*R* ^2^	0.9061
Adj‐*R* ^2^		0.0552	Adj‐*R* ^2^	0.5623	Adj‐*R^2^*	0.9868	Adj‐*R* ^2^	0.7848	Adj‐*R* ^2^	0.8890
Pred‐*R* ^2^		−1.377	Pred‐*R* ^2^	0.3271	Pred‐*R^2^*	0.9577	Pred‐*R* ^2^	0.7158	Pred‐*R* ^2^	0.8335
Adeq.Prec.		2.8361	Adeq.Prec.	7.6889	Adeq.Prec.	38.4643	Adeq.Prec.	11.9147	Adeq.Prec.	17.6553

*Note: X*_1_: CD‐RBLW (clarified and decolorized red beet liquid waste), *X*_2_: 42 DE glucose syrup, *X*_3_: gelatin, *R* ^2^: Coefficient of determination, Adj‐*R* ^2^: Adjusted coefficient of determination, Pred‐*R* ^2^: Predicted coefficient of determination.

Abbreviations: Adeq.Prec, adequate precision; Lof, lack of fit; P.E: pure error; SS, sum of squares.

*p* < 0.05.

The moisture content of samples is critically important due to its effect on major quality characteristics (Ergun et al. 2010; Hartel et al. 2018). Interactions between moisture content and gelatin affect the texture (Dalabasmaz et al. 2023). There is also a significant relationship between the sugar profile of the candy and its moisture content (Ergun et al. 2010). This relationship is primarily on the basis of the varying levels of hydrophilicity of sugars. The moisture content of samples containing CD‐RBLW was above the lower limit for gummy (Hartel et al. 2018) but varied widely (10.71–20.19 g/100 g). It was determined that the effect of independent variables was significant and that the model for these effects had a high *R* ^2^ value (Table 3). Notably, increasing the amount of CD‐RBLW resulted in gummy with higher moisture content. The effect of the gelatin amount was found to be negligible. This result is directly related to the sugar profile of CD‐RBLW, where an increase in fructose concentration resulted in more hydrophilic monosaccharides in the composition. Therefore, the use of CD‐RBLW in gummy candy may affect the crystallization behavior of sucrose (Hartel et al. 2018), the effects of the hydrocolloid gelling agent (de Avelar and Efraim 2020), and especially moisture migration during storage, thereby impacting shelf life (Ergun et al. 2010). In addition to moisture content, water activity (*aw*) is another important physicochemical property of gummy candies. For gummy candies, *aw* values generally range from 0.60 to 0.80, with lower *aw* values resulting in firmer candies (Hartel et al. 2018). In this study, the *aw* values for the samples were between 0.660 and 0.690 (Table 2). Therefore, results consistent with previous studies were obtained (Gok et al. 2020; Kurt et al. 2022; Wang and Hartel 2022). Although a significant linear model was identified for the effect of independent variables on *aw*, this model's low *R* ^2^ value (0.6296) should be noted (Tables 2 and 3). The *aw* values, which varied within a narrow range, were observed to change in parallel with the CD‐RBLW ratio (Supporting Information File 2). However, the results indicate that the tendency for moisture migration or moisture absorption from the environment, which can be associated with *aw*, can be neglected. Although the use of CD‐RBLW caused an increase in the amount of free water, this effect can be attributed to the higher initial total soluble solids (TSS) value of this material compared to glucose syrup. However, the narrow range of variation should be considered. Even though the *aw* values are above 0.600, exceeding the safe level (Fan et al. 2017), modifications to achieve lower values could negatively affect the quality characteristics of gummies, especially texture. Additionally, there is a strong interaction between *aw* values and production technology and composition for candies. In foods with gel structures, sugars affect the matrix's *aw* values (Su et al. 2021). CD‐RBLW modified the gummy samples’ sugar profile, influencing their water activity accordingly. Generally, the presence of sugars leads to a decrease in *aw* values and an increase in the structural stability and melting temperatures of gels (Wang and Hartel 2022). However, these effects may vary depending on the sugar profile and concentration (Pekdogan Goztok et al. 2022).

#### 3.3.2 Color

In confectionery technology, color properties, texture characteristics, and moisture content are crucial for evaluating product stability. There is a relationship between the visual characteristics of candies and the intensity of aroma perceived during consumption (Gunes et al. 2022; Konar et al. 2022). The *L* ^*^ (79.6–97.9), *a* ^*^ [(−1.64)–(−0.45)], *b* ^*^ (4.81–11.0), chroma (4.92–11.1), and hue angle (97.5–100.0) values of the gummy samples are given in Table 3. Significant models related to the effects of independent variables were identified for all samples except for the hue angle, with *R* ^2^ values ranging from 0.7790 to 0.9165 (Table 5). It was particularly noted that the interactions of CD‐RBLW × glucose syrup, CD‐RBLW × gelatin, and glucose syrup × gelatin were significant for the *L* ^*^ value (*p* < 0.05). Using CD‐RBLW increased the *L* ^*^ value of the gummy samples while decreasing the *a* ^*^ and *b* ^*^ values (Supporting Information File 2). However, the fact that the *L* ^*^ values are much higher compared to gummy samples prepared with different colorants or pigment sources is considered a positive finding for the use of CD‐RBLW as a neutral component in terms of color properties (Gok et al. 2020; Kurt et al. 2022). The *L* ^*^, *a* ^*^, *b* ^*^, chroma, and hue angle values of CD‐RBLW samples were 76.6 ± 2.32, −1.71 ± 0.90, 52.9 ± 7.12, and 52.8 ± 7.10, respectively. For commercial glucose syrup, these values were determined as 27.9 ± 0.11, 0.04 ± 0.02, 0.52 ± 0.04, 0.52 ± 0.03, and 85.6 ± 2.78, respectively. Although CD‐RBLW has a significantly higher *L* ^*^ value than glucose syrup, which could be used as an alternative, the high *b* ^*^ values can be considered a disadvantage. Because, in candy production, non‐colorant components should minimally affect visual properties to ensure a neutral background for optimal colorant application (Hubbermann 2016).

**TABLE 5:** Analysis of variance (ANOVA) results for color properties of gummy samples made using clarified and deionized RBLW in different formulations.

	*L* ^*^		*a* ^*^		*b* ^*^		Chroma		Hue angle	
	SS	*p* value	SS	*p value*	SS	*p* value	SS	*p* value	SS	*p* value
Model	905.48	0.0004	1.03	0.0161	46.46	0.0022	46.97	0.0022	8.82	0.3669
*X*_1_ *X*_2_	100.28	0.0143	0.1863	0.0536	2.62	0.1210	2.66	0.1185	1.24	0.747
*X*_1_ *X*_3_	144.95	0.0056	0.0525	0.2644	1.90	0.1783	1.82	0.1865	1.68	0.3055
*X*_2_ *X*_3_	144.44	0.0057	0.028	0.2632	1.94	0.1736	1.87	0.1813	1.75	0.2968
Lof*	80.35	0.0138	0.2458	0.1822	1.74	0.9428	1.77	0.9402	2.34	0.9625
P.E	2.13		0.0458		5.23		5.19		8.87	
Total	987.15		1.32		53.42		53.63		20.04	
*R* ^2^		0.9165	*R* ^2^	0.7790	*R* ^2^	0.8696	*R* ^2^	0.8762	*R* ^2^	0.4404
Adj‐*R* ^2^		0.8643	Adj‐*R* ^2^	0.6409	Adj‐*R* ^2^	0.7882	Adj‐*R* ^2^	0.7891	Adj‐*R* ^2^	0.0906
Pred‐*R* ^2^		0.5168	Pred‐*R* ^2^	0.4682	Pred‐*R* ^2^	0.5499	Pred‐*R* ^2^	0.5508	Pred‐*R* ^2^	−0.9367
Adeq.Prec.		11.3462	Adeq.Prec.	6.3149	Adeq.Prec.	9.2076	Adeq.Prec.	9.1637	Adeq.Prec.	3.3913

*Note: X*_1_: CD‐RBLW (clarified and decolorized red beet liquid waste), *X*_2_:42 DE glucose syrup, *X*_3_: gelatin,, *R* ^2^: Coefficient of determination, Adj‐*R* ^2^: Adjusted coefficient of determination, Pred‐*R* ^2^: Predicted coefficient of determination.

Abbreviations: Adeq.Prec, adequate precision; Lof, lack of fit; P.E: pure error; SS, sum of squares.

*p* < 0.05.

Generally, the results confirmed the effectiveness of applying ion exchange and adsorber resin treatments to CD‐RBLW to remove residual pigments. Visual properties of the powder materials obtained by drying CD‐RBLW without these treatments showed the effect of residual pigments, resulting in samples with relatively high *a* ^*^ values (Atalar et al. 2024). The primary reason for this is the high content of red‐violet‐colored betaine in the waste products following the RB extract process (Nemzer et al. 2011). The findings from our study, along with the near‐zero *a* ^*^ values of the samples, may also be influenced by the instability of residual pigments. This is because thermal processing was applied to prepare gummy samples, and residual betalains can degrade during this process. These pigments are highly sensitive to heat and oxidation, undergoing isomerization and decarboxylation reactions under such environmental conditions (Herbach et al. 2006). Generally, in the production of gelatin‐containing gummy candies, thermal processing causes a decrease in *L* ^*^ values and increases in *a* ^*^ and *b* ^*^ values (Wang and Hartel 2022). However, in this study, relatively high *L* ^*^ values were obtained.

#### 3.3.3 Total Phenolic Content

RB and its products are plant materials rich in polyphenols, especially due to their phenolic acids and flavonoids (Costa et al. 2017; Ravichandran et al. 2013). In previous studies, the TPC value for RB juice was determined to be 590.0 mg/L (Trishitman et al. 2021). Atalar et al. (2024) reported TPC values of 11.4–257.4 mg GAE/kg for CD‐RBLW dried with different ratios of maltodextrin. Considering the maltodextrin ratio used in the study (10%–25%), it can be stated that the TPC values of CD‐RBLW are consistent with the TPC value of RB juice. In this study, the total phenolic content of CD‐RBLW samples after clarification and color removal was 58.1 ± 0.83 mg GAE/kg. In the gummy samples, the TPC was determined to be 5.33 ± 0.10–45.65 mg GAE/kg (Table 3). Therefore, it can be stated that the polyphenols present in CD‐RBLW did not suffer significant loss under the environmental conditions encountered during the preparation of the gummy samples. Generally, TPC values of gummy samples were negligible. For this reason, using this alternative and innovative material did not have the potential to enrich the gummy's bioactive component composition. However, some factors need to be considered. One of these is the potential effects of polyphenol‐protein interactions on the bioavailability properties of polyphenols. Gelatin, a hydrocolloid protein, was used in the gummy composition. Reversible and irreversible protein‐polyphenol interactions can negatively impact the delivery of these bioactive components to target tissues and cells and potentially affect their physiological activities (Gallo et al. 2013; Rohn 2014). Therefore, the increased TPC due to CD‐RBLW may have lower utilization and physiological effects after gummy consumption due to the presence of gelatin in the composition. Another issue is related to the effect of this interaction on the gel's structural properties. Polyphenols can bind at different points on the protein chain (Dalabasmaz et al. 2023). As a result of these bindings, the number of sucrose binding sites and gelatin‐gelatin bonds necessary for the formation of the gelatin network structure may decrease. This situation can affect the gel's structural properties. Therefore, although the increase in TPC value has potentially positive effects on nutrition and health, it may have adverse effects regarding techno‐functional properties. Consequently, it would be beneficial to develop and apply additional processes and/or methods to reduce the polyphenol concentration during the production of CD‐RBLW. Additionally, it would be useful to examine the effects on texture properties, considering these potential impacts and interactions. In this study, a significant model was determined for the TPC values of the gummy samples. The *R* ^2^ value of this linear model was 0.8179. As predicted, it was found that the TPC value increased with the increase in the amount of CD‐RBLW used (Supporting Information File 2).

#### 3.3.4 Texture

Gummy producers have to adjust the textural parameters of their products in line with consumer expectations. Textural properties of gummies affect taste and aroma release. For this purpose, many parameters, such as the amount of ingredients type and concentration, and production conditions affect the textural properties. For this purpose, it is important to monitor the textural stability from production until the shelf‐life process. The primary texture properties of the samples, including hardness (1016.3–27,776.9 g), springiness (30.6%–69.37%), cohesiveness (62.7%–97.93%), gumminess (71,793–270,629 g), chewiness (22,744–176,658 g), and resilience (0.121–0.868), were examined (Table 6). Significant linear models were determined for all properties except springiness concerning the effects of the independent variables (*p* < 0.05). The *R* ^2^ values of these models ranged from 0.8032 to 0.8754 (Table 7). For all texture properties with significant models, notable interactions were observed between glucose syrup and CD‐RBLW, as well as between glucose syrup and gelatin. Additionally, for hardness and gumminess, the interaction between CD‐RBLW and gelatin was significant (*p* < 0.05). Previous studies have identified different TPA values for gummy samples. The possible reasons for this include the strong relationship between texture properties and numerous quality characteristics, the reproducibility of TPA, the geometry and physical properties of the samples, the type and amount of hydrocolloid used, and the conditions for hydrocolloid addition. Therefore, findings should be compared and discussed within their context, particularly in reformulation and component substitution studies. Our study also examined variations in TPA parameters due to different variables and how texture properties responded to these composition factors. Hardness is a key texture parameter in confectionery classification, influenced by hydrocolloid type and concentration, processing conditions, and ingredient composition (Dalabasmaz et al. 2023; Wang and Hartel 2022). Changes that affect the structure formed by hydrocolloids in the product composition result in gummies and gel structures with different hardness properties (Aidat et al. 2023). Another factor that should be considered is the changes in gelation behavior depending on the presence and amount of polyphenols. This is because interactions between these compounds and gelatin could influence the gelatin cross‐linking behavior. Findings from a recent study also support this possibility (Atalar et al. 2025). In future studies, it would be beneficial to investigate these interactions for CD‐RBLW.

**TABLE 6:** Texture properties of gummy samples made using clarified and decolorized red beet liquid waste (CD‐RBLW) in different formulations.

Sample	Hardness	Springiness	Cohesiveness	Gumminess	Chewiness	Resilience
1	1506.42 ± 29.34	19.76 ± 5.84	97.72 ± 0.29	147,206.95 ± 3294.65	24,458.07 ± 1497.94	82.69 ± 1.34
2	1480.94 ± 54.85	54.22 ± 3.42	97.76 ± 0.98	144,763.5 ± 4905.13	78,383.35 ± 2528.22	77.82 ± 1.12
3	1158.96 ± 130.65	69.37 ± 6.95	93.6 ± 0.99	108,471.65 ± 12,228.68	74,872.55 ± 6551.23	55.17 ± 1.87
4	1150.99 ± 60.58	49.21 ± 2.86	95.11 ± 1.56	109,533.2 ± 7591.1	53,761.68 ± 733.08	55.55 ± 3.69
5	2389.68 ± 101.63	64.81 ± 4.12	88.34 ± 3.27	210,926.72 ± 1167.7	136,669.87 ± 7926.6	42.46 ± 4.21
6	2776.92 ± 169.77	65.43 ± 4.77	97.45 ± 0.16	270,629.07 ± 16,981.42	176,658.47 ± 1791.45	84.68 ± 0.26
7	1016.3 ± 59.35	54.83 ± 12.63	80.69 ± 5.13	82,013.88 ± 7364.25	45,046.94 ± 11,036.42	21.77 ± 3.05
8	1174.88 ± 334.48	31.65 ± 1.09	62.7 ± 9.05	71,793.52 ± 10,665.61	22,744.6 ± 3650.62	12.12 ± 2.75
9	1352.42 ± 47.04	41.48 ± 1.89	92.47 ± 1.56	116,745.2 ± 12,638.25	48,368.63 ± 4920.72	46.25 ± 1.15
10	1202.02 ± 22.58	38.71 ± 4.2	94.64 ± 2.37	113,800.45 ± 4999.36	43,991.47 ± 4316.55	51.9 ± 7.82
11	1945.6 ± 368.42	33.34 ± 4.18	97.75 ± 0.66	190,069.47 ± 34,725.67	62,649.13 ± 3641.29	79.05 ± 0.01
12	1272.28 ± 102.03	30.6 ± 2.96	97.39 ± 0.14	123,913.06 ± 9986.91	37,796.02 ± 2921.92	77.37 ± 0.86
13	1100.47 ± 84.85	34.57 ± 5.9	97.93 ± 0.21	107,779.09 ± 8545.54	37,147.1 ± 2418.59	75.11 ± 3.23
14	1710.18 ± 14.4	41.61 ± 0.23	97.75 ± 0.14	164,961.48 ± 4783.49	69,044.52 ± 1799.83	86.81 ± 0.94

*Note*: Mean ± standard deviation.

**TABLE 7:** Analysis of variance (ANOVA) results for texture properties of gummy samples made using clarified and decolorized red beet liquid waste (CD‐RBLW) in different formulations.

	Hardness (g)		Resilience (%)		Cohesion (%)		Gumminess (g)		Chewiness (g)		Springiness (%)	
	SS	*p* value	SS	*p* value	SS	*p* value	SS	*p* value	SS	*p* value	SS	*p* value
Model	2.88E + 06	0.0094	5947.44	0.0001	1027.96	0.0018	3.17E + 10	0.0048	2.11E + 10	0.0069	nd	nd
L. Karışım	1.54E + 06	0.0089	5947.44	0.0001	725.59	0.0013	2.10E + 10	0.0025	8.09E + 09	0.0103		nd
*X*_1_ *X*_2_	8.88E + 05	0.0121	–	–	166.48	0.0274	5.64E + 09	0.0257	1.83E + 09	0.0775	nd	nd
*X*_1_ *X*_3_	6.94E + 05	0.0214	–	–	–	–	7.13E + 09	0.0153	7.68E + 07	0.6849	nd	nd
*X*_2_ *X*_3_	6.89E + 05	0.0217	–	–	98.17	0.0742	7.11E + 09	0.0153	9.01E + 07	0.6606	nd	nd
*X*_1_ *X*_2_ *X*_3_									6.01E + 09	0.0072	nd	nd
Lof*	6.668E + 05	0.0105	1200.29	0.3506	45.51	0.4096	5.98E + 09	0.003	5.87E + 08	0.9332	nd	nd
P.E	14,608.3		257.25		171.24		5.55E + 07		2.41E + 09		nd	
Total	3.56E + 06		7404.98		1244.72		3.78E + 10		2.41E + 10		nd	
*R* ^2^ Adj‐*R* ^2^ Pred‐*R* ^2^ Adeq.Prec.		0.8086	*R* ^2^	0.8032	*R* ^2^	0.8259	*R* ^2^	0.8401	*R* ^2^	0.8754	*R* ^2^	nd
		0.689	Adj‐*R* ^2^	0.7674	Adj‐*R* ^2^	0.7485	Adj‐*R* ^2^	0.7401	Adj‐*R* ^2^	0.7687	Adj‐*R* ^2^	nd
		−0.0986	Pred‐*R* ^2^	0.6634	Pred‐*R* ^2^	0.3918	Pred‐*R* ^2^	0.0431	Pred‐*R* ^2^	0.5551	Pred‐*R* ^2^	nd
		8.3901	Adeq.Prec	12.552	Adeq.Prec	8.8732	Adeq.Prec	9.6037	Adeq.Prec	9.8006	Adeq.Prec	nd

*Note: X*_1_: CD‐RBLW (clarified and decolorized red beet liquid waste), *X*_2_:42 DE glucose syrup, *X*_3_: gelatin. *R* ^2^: coefficient of determination, Adj‐*R* ^2^: adjusted coefficient of determination, Pred‐*R* ^2^: predicted coefficient of determination.

Abbreviations: Adeq.Prec, adequate precision; Lof, lack of fit; P.E: pure error; SS, sum of squares.

*p* < 0.05.

As expected, in our study, the increase in gelatin amount resulted in increased hardness. Additionally, higher amounts of CD‐RBLW led to a harder gummy structure. It can be anticipated that CD‐RBLW, which contains more monosaccharides than glucose syrup, including fructose characterized by high moisture affinity (Ergun et al. 2010), would produce a less hard gummy. However, the presence of sucrose, another major component of CD‐RBLW, should also be considered. The presence and amount of sucrose in the matrix support the binding points of the gelatin network structures, resulting in a stronger gel formation. Although the fructose in CD‐RBLW could reduce hardness, its sucrose content contributed to a firmer structure. Additionally, the impact of CD‐RBLW on pH should be considered in evaluating these results, as there is a strong relationship between pH value during thermal processing and gelatin bloom value losses (Hartel et al. 2018). The cohesiveness values of the gummy samples ranged from 62.7% to 97.93%, generally above 94% (Table 6). This parameter typically does not correlate with other TPA parameters. As cohesiveness indicates the strength of internal bonds, it should be considered separately from other parameters. Lower glucose syrup usage resulted in lower cohesiveness. Additionally, increased CD‐RBLW and gelatin amounts led to higher cohesiveness. This result aligns with the gelatin concentration‐cohesiveness relationship highlighted by Wang and Hartel (2022). The interaction between gelatin concentration and CD‐RBLW suggests that this innovative ingredient influences gelatin cross‐linking due to its distinctive sugar composition (Amjadi et al. 2018). Compositional factors also influence the mechanical properties of gel structures. Increases in gelatin concentration and the presence and concentration of sugars enhance the strength of the gelatin network in water (Ge et al. 2021; Xie et al. 2023). In previous studies, whereas an increase in hardness values was observed for gel‐structured foods, significant changes in cohesiveness and springiness values were not determined (Zhang et al. 2023). However, our study observed changes not only in hardness but also in other values. The springiness values for gummy samples containing CD‐RBLW showed a wide range of variation (19.76%–65.43%). Springiness is related to TPA parameters such as chewiness and hardness. Additionally, springiness values are used for some products as indicators of air‐holding capacity (Zhao et al. 2021). However, our study determined no significant effect of independent variables on springiness. Chewiness is a TPA parameter strongly correlated with sensory properties. It is directly related to the responses produced by the product in the oral cavity and during mastication. It is associated with the energy required for chewing soft and semi‐solid foods (Gok et al. 2020). Therefore, higher chewiness values are observed for gummies with higher hardness and lower springiness. In other words, increased hardness generally leads to higher chewiness values (Aidat et al. 2023). Some studies suggest characterizing gummi and jelly candies on the basis of chewiness values rather than gumminess (Delgado and Bañón 2018), arguing that these confections typically have a solid matrix. However, our study did not yield similar results for chewiness concerning the effects of CD‐RBLW and glucose syrup concentrations on hardness. In particular, gelatin concentration and its interactions with other independent variables impact chewiness significantly. These findings align with observations on the relationship between gelatin concentration, chewiness, and gumminess values when developing confections with different sugar alternatives (Periche et al. 2014). Gumminess was also examined in our study as a texture parameter generally associated with the hardness of gel structures. Similar findings were obtained in our study, indicating that increased CD‐RBLW concentration led to increased hardness and gumminess values in gummy samples. Furthermore, significant models associated with the effects of independent variables on both gumminess and chewiness values were identified, highlighting the notable interactions between these parameters (*p* < 0.05). In our study, another TPA parameter examined is resilience, which is used to assess the recovery behavior of the gel structure after applying force (Wu et al. 2023). Previous research has shown that different processing conditions (Dalabasmaz et al. 2023), properties related to gelatin (Ge et al. 2021), and components, including sugars (Wang and Hartel 2022), affect the resilience of gel structures. Similar effects have been observed with CD‐RBLW, as our study found that CD‐RBLW in gummy samples increased resilience values. However, it was determined that the intensity of this increase was enhanced with increasing gelatin concentration.

#### 3.3.5 FT‐IR Spectra

**FIGURE 1:**
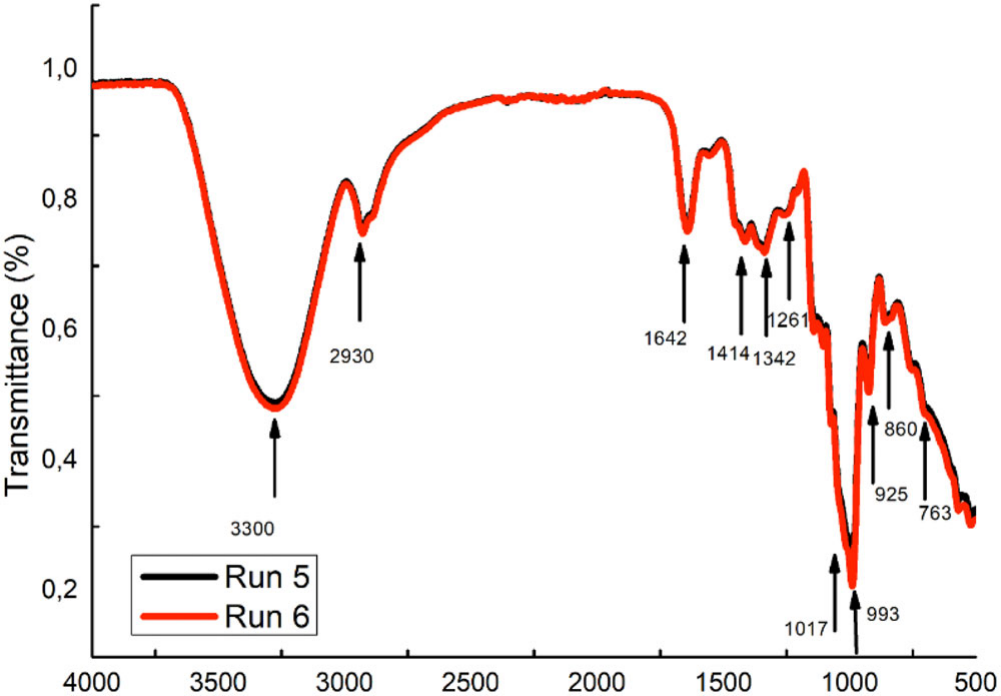
FT‐IR spectra of gummy Samples 5 and 6. Run 5: 0 g/100 g CD‐RBLW, 39.70 g/100 g glucose syrup, 7 g/100 g gelatine; Run 6: 39.70 g/100 g CD‐RBLW, 0 g/100 g glucose syrup, 7 g/100 g gelatine.

FT‐IR spectroscopy monitors the structural changes in the samples. In Figure 1, FT‐IR spectroscopy was used to analyze the effects of varying glucose syrup and CD‐RBLW concentrations in the formulation. The band around 3300 cm^−1^ is mainly related to the OH stretching vibrations of water. The intensity of this band was enhanced with the use of CD‐RBLW. The peaks around 2930 cm^−1^ correspond to the C–H deformation vibration of carboxylic acids and NH_3_ stretching vibrations of free amino acids. The most significant band (Amide I) related to the gelatin is observed in the 1700–1600 cm^−1^ spectral range (Cebi et al. 2019). Gummy contains sugar (sucrose) and corn syrup; therefore, it is expected to see sugar‐based peaks in wavenumbers between 1500 and 750 cm^−1^. The peaks at 925, 1261, and 1414 cm^−1^ can be attributed to the C–H bending, C–O stretching, and O–H stretching/bending vibrations of bending modes of CH_2_ and CH_3_ groups in proteins and carbohydrates, respectively (Tewari and Irudayaraj 2004). These bands’ intensity was increased by using CD‐RBLW to formulate gummy samples. The band observed around 993 cm^−1^ corresponds to the vibrational band of glycosidic links of sucrose. The peak around 925 cm^−1^ belongs to the α‐anomeric linkage between glucose and fructose in sucrose (Nizamlioglu et al. 2022). The band observed around 860 cm^−1^ is mainly related to the fructose. The highest transmittance values in sugar‐based peaks were obtained from CD‐RBLW used gummy formulations.

#### 3.3.6 Accelerated Shelf Life

Due to their relatively low water activity and high sugar concentrations, the susceptibility of gummies to microbiological spoilage, like most other confectionery products, can be considered negligible. Consequently, shelf‐life studies generally do not include microbial spoilage (Subramaniam 2016). For this reason, gummy samples were stored under ASL conditions for 8 weeks (25°C/70% RH), and changes in texture properties, especially hardness, color difference (∆*E*), and water activity values, were monitored periodically under these conditions (Table 8). Replacing glucose syrup with CD‐RBLW led to a slower and less pronounced change in hardness. This effect is primarily attributed to differences in sugar profiles. A similar interaction was observed for water activity values. For these reasons, it can be stated that using CD‐RBLW improved gummy stability.

**TABLE 8:** Zero‐order rate constant under accelerated shelf‐life conditions for water activity values of gummy samples made using clarified and decolorized red beet liquid waste (CD‐RBLW) in different formulations.

RBLW (g/100 g, in dm)	Glucose syrup (g/100 g, in dm)	Gelatin, (g/100 g, in dm)	Water activity (aw)			Color difference (∆*E*)			Hardness		
			∆*aw* (%)	*k*_0_	*R* ^2^	∆*E*_Day49_	*k*_0_	*R* ^2^	∆Hardness (%)	*k*_0_	*R* ^2^
41.00	0.00	5.70	5.71	0.0011	0.8245	12.4	0.1819	0.7432	16.1	64.4	0.6037
41.00	0.00	5.70	7.12	0.0017	0.9735	14.9	0.1991	0.7534	18.1	57.4	0.5836
20.13	20.13	6.45	12.1	0.0018	0.9164	6.27	0.1019	0.8471	90.3	124.5	0.8249
27.57	14.13	5.00	10.6	0.0017	0.8488	8.23	0.1685	0.9024	85.1	143.7	0.8766
0.00	39.70	7.00	12.1	0.0018	0.8507	3.29	0.0581	0.7653	45.1	153.3	0.7417
39.70	0.00	7.00	8,06	0.0012	0.7493	18.5	0.2778	0.8421	4.1	47.7	0.1302
0.23	41.00	5.47	14.2	0.0019	0.9547	6.11	0.0929	0.7894	168.1	279.2	0.9379
0.23	41.00	5.47	10.4	0.0016	0.8655	8.77	0.1289	0.5532	141.9	259.3	0.8499
10.20	30.05	6.45	10.0	0.0014	0.8397	8.31	0.0638	0.7727	65.2	161.8	0.7800
13.23	26.47	7.00	12.4	0.0017	0.8999	9.50	0.0748	0.7688	118.7	240.2	0.8883
26.47	13.23	7.00	9.26	0.0015	0.8568	9.07	0.0690	0.7999	5.1	39.8	0.4604
20.85	20.85	5.00	5.70	0.0011	0.7543	17.3	0.2653	0.5830	56.2	114.2	0.9142
20.13	20.13	6.45	7.97	0.0015	0.8610	12.8	0.1951	0.7404	64.4	122.3	0.6092
30.05	10.20	6.45	7.60	0.0014	0.8551	15.4	0.2462	0.7175	25.1	73.6	0.9922

According to ASL findings, CD‐RBLW was generally disadvantageous for color stability, as results were above the accepted limit value (Δ*E* > 3.0) for visually perceptible color difference. Betalains are sensitive to temperature and oxidation, undergoing degradation due to isomerization and decarboxylation during heating processes (Herbach et al. 2006). Factors such as a decrease in pH and an increase in temperature accelerate betalain degradation rates (Trishitman et al. 2021), with the optimum pH range being determined as 4–6. In food compositions utilizing betalains, monitoring *a* ^*^ values can assess the stability of these pigments (Chandran et al. 2014).

## 4 Conclusion

This study validates the potential of a waste by‐product as an alternative ingredient in gummy candy formulations, offering cost‐effective waste management and improved environmental sustainability. Modifying visual properties derived from residual pigments of RBLW increases its potential for use in food compositions. Considering its intended use, removing specific macro‐ and micro‐components is crucial for enhancing its applicability in food formulations, particularly in confectionery technologies where reducing ash content, residual pigments, polyphenols, and raw protein levels can improve its utility. However, its use as a sugar alternative does not focus on reducing calorie levels or developing low‐sugar products. Following clarification and decolorization, RBLW can be recycled and evaluated as an alternative food ingredient, especially in high‐usage products. Consideration should also be given to its interaction with other components, particularly gelatin, and its impact on critical quality attributes and shelf‐life stability of confectionery products. Its lower pH than glucose syrup is also significant for the aforementioned effects. Future reformulation efforts should aim to optimize CD‐RBLW's TSS content to broaden its applications. Moreover, CD‐RBLW enhances its potential not only as a substitute for monosaccharide sources but also for partial substitution of disaccharides. The results indicated that RBLW, after clarification and decolorization, could be used in the confectionery industry and that the waste from the natural colorant industry can be utilized as innovative food ingredients. This process offers innovative approaches in the food industry that align with the principles of sustainability and circular economy.

RBLW provides a sustainable alternative to traditional glucose syrup in food formulations, particularly for gummies. After processing, RBLW demonstrated similar functionality to glucose syrup, maintaining comparable textural properties, moisture retention, and shelf‐life stability in gummies. The decolorization process ensures that the product's visual properties remain unaffected. From a sustainability standpoint, RBLW helps mitigate waste from RB pigment extraction, contributing to a circular economy by repurposing by‐products that would otherwise create disposal challenges. This solution reduces environmental impact, lowers dependence on conventional crops like corn, and promotes resource efficiency, positioning RBLW as a more eco‐friendly and efficient option than glucose syrup.

Additionally, it is necessary to mention some limitations of this study. The lack of sensory analyses in the gummy samples, the failure to examine the relationship between aroma release behavior and the use of CD‐RBLW using chromatographic techniques in these samples, and the laboratory‐scale studies conducted in the development of the CD‐RBLW process are the main limitations. In future studies, toxicological investigations of CD‐RBLW, along with the validation of pilot‐scale clarification and decolorization processes before industrial‐scale applications, are required. These scale‐up studies will not only further reduce protein and total ash content but also increase the T625 value for clarity above 95%, enhancing the potential for the industrial use of CD‐RBLW.

## Author Contributions

**Tahra Elobeid**: writing–review and editing. **Burcu Tüzün**: formal analysis. **Ayşe Apaydın**: writing–review and editing. **Ezgi Tekneci**: formal analysis. **Ibrahim Palabiyik**: writing–review and editing, writing–original draft. **Omer Said Toker**: writing–review and editing. **Nevzat Konar**: writing–review and editing. **İlyas Atalar**: conceptualization, methodology, supervision, writing–review and editing.

## Conflicts of Interest

The authors declare no conflicts of interest.

## Supporting information



Supporting Information
