# Supplementary material for: Red Beet Process Waste: A Sustainable Glucose Syrup Alternative for Gummy Confectionery
Source: J Food Sci. 2025 May 7;90(5):e70262. doi: 10.1111/1750-3841.70262 (PMC12057549; doi:10.1111/1750-3841.70262)
Supplement: Supplementary file 1 — Supporting Information [file JFDS-90-0-s001.docx]

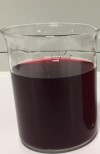

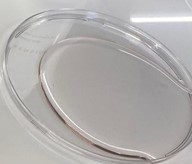


**Supplementary File 1.** Red beet process liquid waste (RBLW) before (a) and after (b) clarification and


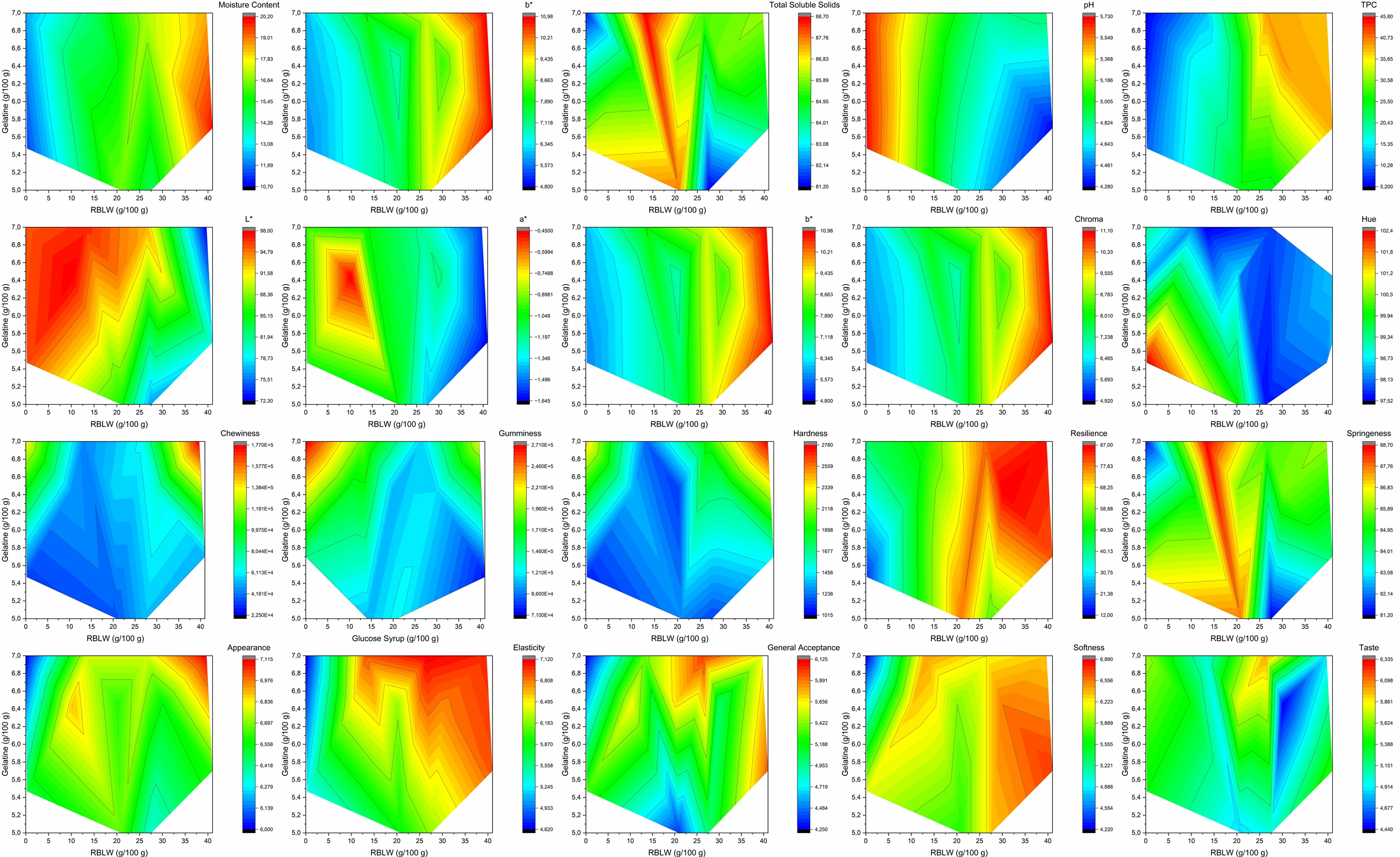


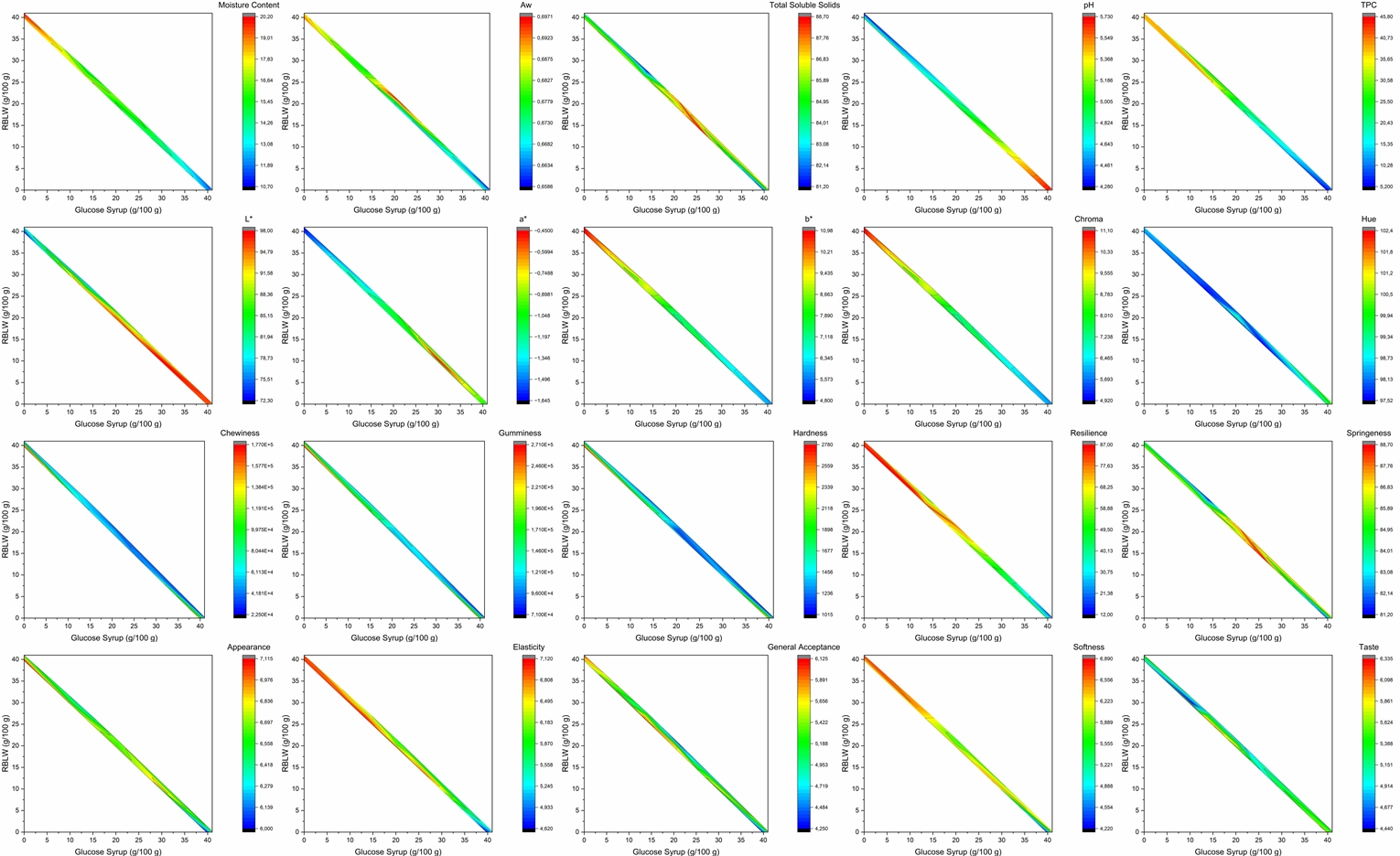


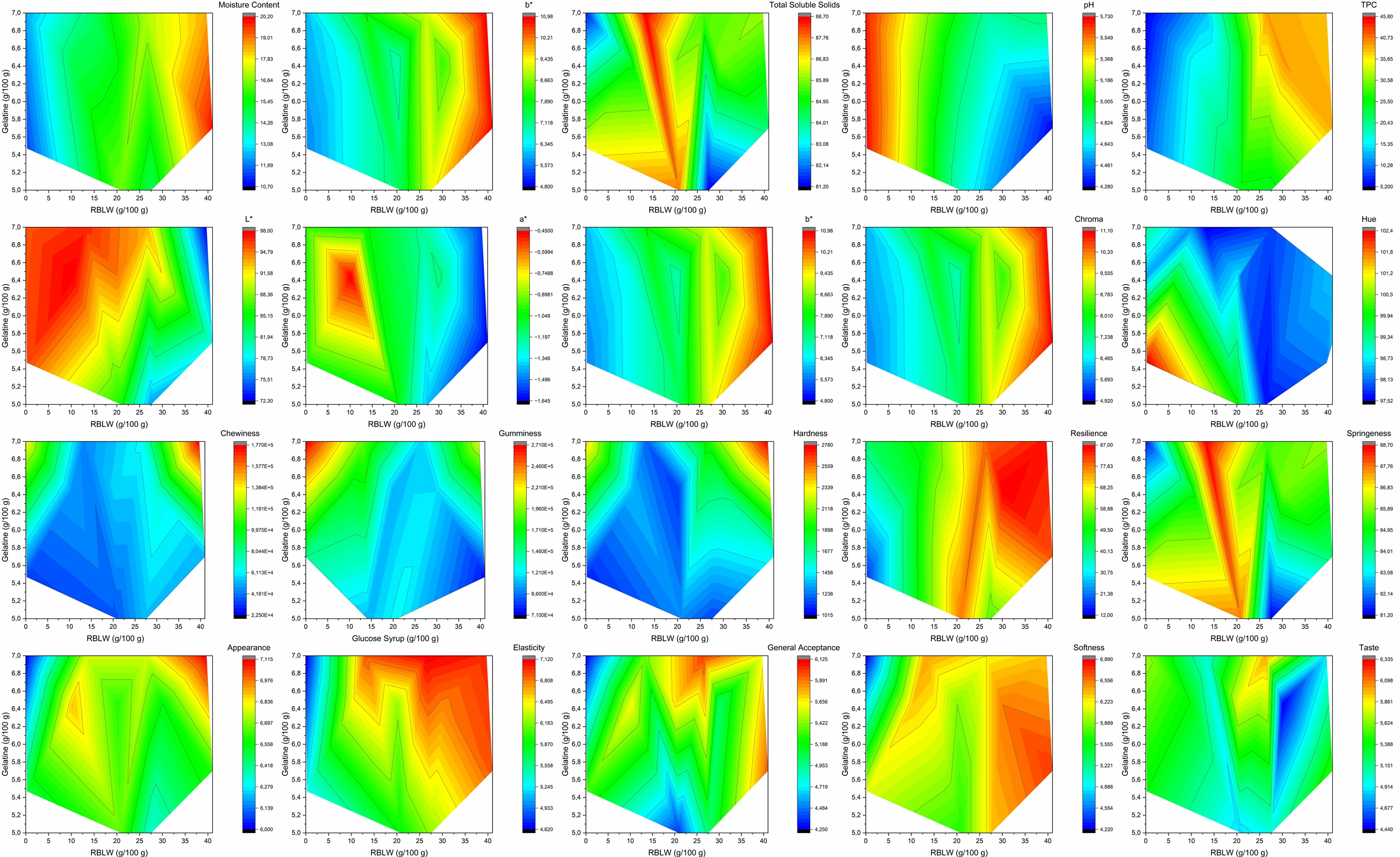


**Supplementary File 2.** Interactions of independent variables (clarified and decolorized red beet coloring agent liquid waste, glucose syrup and gelatine) on various quality parameters of gummy samples
